# Predictive Value of Blood Coagulation Parameters in Poor Outcomes in COVID-19 Patients: A Retrospective Observational Study in Romania

**DOI:** 10.3390/jcm11102831

**Published:** 2022-05-17

**Authors:** Cosmin Citu, Bogdan Burlea, Florin Gorun, Andrei Motoc, Oana Maria Gorun, Daniel Malita, Adrian Ratiu, Roxana Margan, Mirela Loredana Grigoras, Felix Bratosin, Ioana Mihaela Citu

**Affiliations:** 1 Department of Obstetrics and Gynecology, “Victor Babes” University of Medicine and Pharmacy, 2 Eftimie Murgu Square, 300041 Timisoara, Romania; citu.ioan@umft.ro (C.C.); ratiu.adrian@umft.ro (A.R.); 2 Department of Obstetrics and Gynecology, Municipal Emergency Clinical Hospital Timisoara, 1–3 Alexandru Odobescu Street, 300202 Timisoara, Romania; bogdanburlea@yahoo.com (B.B.); oanabalan@hotmail.com (O.M.G.); 3 Department of Anatomy and Embryology, “Victor Babes” University of Medicine and Pharmacy, 2 Eftimie Murgu Square, 300041 Timisoara, Romania; amotoc@umft.ro (A.M.); grigoras.mirela@umft.ro (M.L.G.); 4 Department of Radiology, “Victor Babes” University of Medicine and Pharmacy, Eftimie Murgu Square nr. 2, 300041 Timisoara, Romania; malita.daniel@umft.ro; 5 Department 14 Microbiology, Discipline of Hygiene, Center for Studies in Preventive Medicine, “Victor Babes” University of Medicine and Pharmacy, 300041 Timisoara, Romania; marganroxana@gmail.com; 6 Methodological and Infectious Diseases Research Center, Department of Infectious Diseases, “Victor Babes” University of Medicine and Pharmacy, 300041 Timisoara, Romania; felix.bratosin7@gmail.com; 7 Department of Internal Medicine I, “Victor Babes” University of Medicine and Pharmacy, 2 Eftimie Murgu Square, 300041 Timisoara, Romania; citu.ioana@umft.ro

**Keywords:** COVID-19, coagulation, prediction

## Abstract

SARS-CoV-2 infection produces alterations in blood clotting, especially in severe cases of COVID-19. Abnormal coagulation parameters in patients with COVID-19 are important prognostic factors of disease severity. The objective of this study was to evaluate the predictive value of aPTT, D-dimer, INR and PT in the mortality of patients with COVID-19. A retrospective, single-center, observational study was conducted on COVID-19 patients admitted to the Municipal Emergency Clinical Hospital in Timisoara, Romania, between August and October 2021. Patients were confirmed as COVID-19 positive by reverse transcription-polymerase chain reaction (RT-PCR) assay. After applying the inclusion/exclusion criteria, a total of 82 patients were included in the analysis. Receiver operating characteristic (ROC) curves of D-Dimer, INR, PT and aPTT were generated to assess whether the baseline of each of these biomarkers was accurately predictive for mortality in patients with COVID-19. Mortality among patients enrolled in this study was 20.7%, associated with older age and presence of heart disease. The areas under the ROC curve (AUC-ROC) of D-Dimer, INR, PT, and aPTT were 0.751, 0.724, 0.706 and 0.753. Differences in survival for patients with coagulation biomarker levels above cut-off values compared to patients below these values were statistically significant. All evaluated parameters had significant differences and good performance in predicting mortality of COVID-19 patients, except fibrinogen, which had no significant difference. Moreover, aPTT and D-dimer were the best performing parameters in predicting mortality in patients with SARS-CoV-2 infection.

## 1. Introduction

COVID-19 (coronavirus disease 2019) is an infectious respiratory disease caused by the severe acute respiratory syndrome coronavirus 2 (SARS-CoV-2). Reacting to the dramatic rises in the incidence of new cases starting in December 2019, the World Health Organization (WHO) declared a pandemic in March 2020. Since then, COVID-19 has been responsible for nearly 6 million deaths and more than 400 million cases in the global population [1,2,3]. In Romania, at the time of writing, more than 2.5 million cases and 62 thousand deaths have been recorded, ranking us 34th globally [3]. COVID-19 is an inflammatory disease characterized by flu-like symptoms, and in severe cases leads to atypical pneumonia which can eventually advance to acute respiratory distress syndrome (ARDS) and demise [4,5]. Following SARS-CoV-2 infection, as with all infections, the human body, to defend itself, naturally triggers the innate immune system, although excessive immune responses can lead to inflammatory storms with injury to the microcirculation and activation of the coagulation system with the development of disseminated intravascular coagulation [6]. A state of hypercoagulability has been identified in cases of COVID-19, being a key aspect of micro- and macro- vascular thrombosis found in patients with this infectious disease [7,8]. Abnormalities of blood coagulation parameters were reported, particularly in patients with COVID-19 associated pneumonia and ARDS [9]. In addition, increased D-dimer values, prolonged prothrombin time (PT) and increased fibrinogen values have been reported in COVID-19 patients. [10,11]. Therefore, the aim of this article is to assess the predictive value of INR (international normalized ratio), APTT (activated partial thromboplastin time), D-dimer and PT in the mortality of Romanian patients with COVID-19.

## 2. Materials and Methods

### 2.1. Study Design and Settings

A retrospective, single-center, observational study was performed on COVID-19 patients who were hospitalized at the Municipal Emergency Hospital in Timisoara, Romania, from August to October 2021. Patients were confirmed as COVID-19 positive by reverse transcription-polymerase chain reaction (RT-PCR) assay.

This study was approved by the Ethics Committee of the Municipal Emergency Clinical Hospital in Timisoara (No. I-32467/23 December 2021).

### 2.2. Participants

The inclusion criteria of the research participants were as follows: (1) patients hospitalized at the Timisoara Municipal Clinical Emergency Hospital between August and October 2021; (2) COVID-19 confirmed according to the guidelines established by the National Centre for Surveillance and Control of Communicable Diseases in Romania, by positive test for SARS-CoV-2 using RT-PCR on a nasopharyngeal swab; (3) complete documentation of coagulation markers taken at admission; (4) age over 18 years. Individuals younger than 18 years old and without complete documentation of laboratory coagulation tests or who were given anticoagulant prior to admission were excluded.

### 2.3. Variables and Data Sources

Demographic elements, clinical data and laboratory evaluations collected at admission were extracted from electronic medical records archived in the database of the Timisoara Municipal Emergency Hospital. Variables consisted of age, gender and comorbidities, coagulation test values (D-Dimer, aPTT, PT, INR and Fibrinogen), duration of hospitalization and admission to the intensive care unit. Data were also collected on the outcome (death or discharged cured)

### 2.4. Statistical Analysis

The statistical analysis was carried out by using RStudio. Continuous variables were presented as median (interquartile range) and compared by Mann–Whitney test. Categorical variables were expressed in count and percentage and were compared by chi-square test. Optimal cut-off values have been obtained using the Youden index. The predictive performance of coagulation biomarkers for COVID-19 mortality was assessed by estimating the area under the curve of the corresponding receptor operating curve. Binomial logistic regression was performed to assess the independent predictive value for all blood coagulation biomarkers.

## 3. Results

### 3.1. Participants Selection and Baseline Characteristics

**Figure 1:**
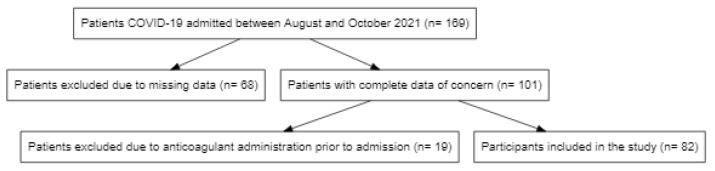
Flowchart of participant selection for the analysis.

Out of a total of 169 COVID-19 patients admitted between August and October 2021 at the Clinical Municipal Emergency Hospital Timisoara, 82 were included in the analysis and were followed up during hospitalization (Figure 1).

Among the patients, the median age (IQR) was 66.5 (17) years, 51.2% of whom were female. The most frequently reported comorbidities were hypertension (64.6%), heart disease (42.7%) and chronic kidney disease (42.7%). The patients who died in hospital were statistically significantly older than the patients in the survivor cohort. There were no statistically significant differences in the frequency of comorbidities between surviving and non-surviving patients, with the exception of heart disease. Regarding coagulation biomarkers, except fibrinogen, there were significantly increased values in the cohort of non-survivors (Table 1).

### 3.2. Coagulation Biomarker Cut-Off Values in Predicting Mortality in Patients with COVID-19

**Figure 2:**
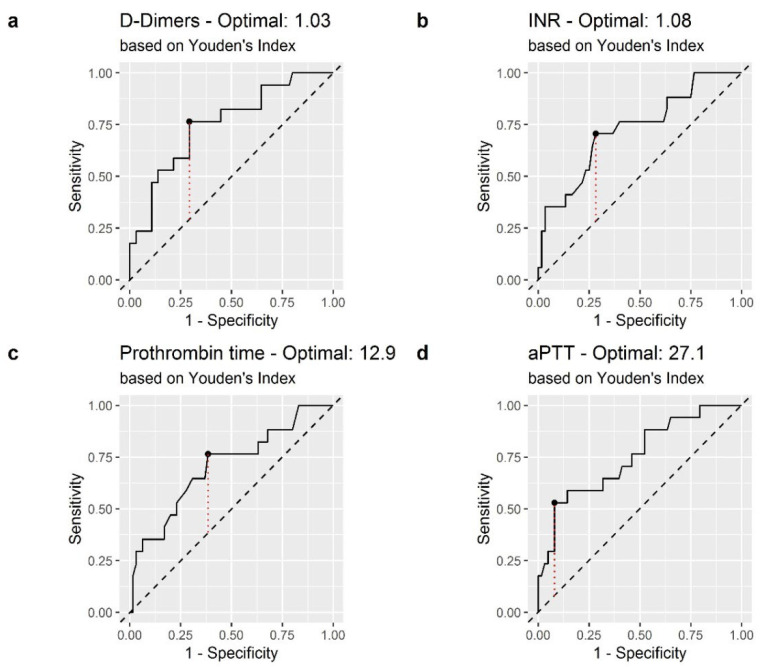
Receiver operating characteristic (ROC) curve of coagulation biomarkers in the prediction of in-hospital mortality: (**a**) D-Dimer; (**b**) International Normalized Ratio (INR); (**c**) Prothrombin Time (PT) (**d**) Activated Partial Thromboplastin Time (aPTT). The dashed red line represents the Youden index.

Receiver operating characteristic (ROC) curves of D-Dimer, INR, PT and aPPT were generated to assess whether the baseline of each of these biomarkers was accurately predictive for mortality in patients with COVID-19 (Figure 2). Fibrinogen had AUC-ROC less than 0.500 (AUC = 0.496), and was excluded from statistical analysis.

The areas under the curve (AUC) of D-Dimer, INT, PT, and aPPT were 0.751, 0.724, 0.706 and 0.753, respectively. The optimal cutoff values obtained from Youden’s index are shown in Table 2.

### 3.3. Association of D-Dimer, INR, PT and aPTT Scores with COVID-19 Mortality

**Figure 3:**
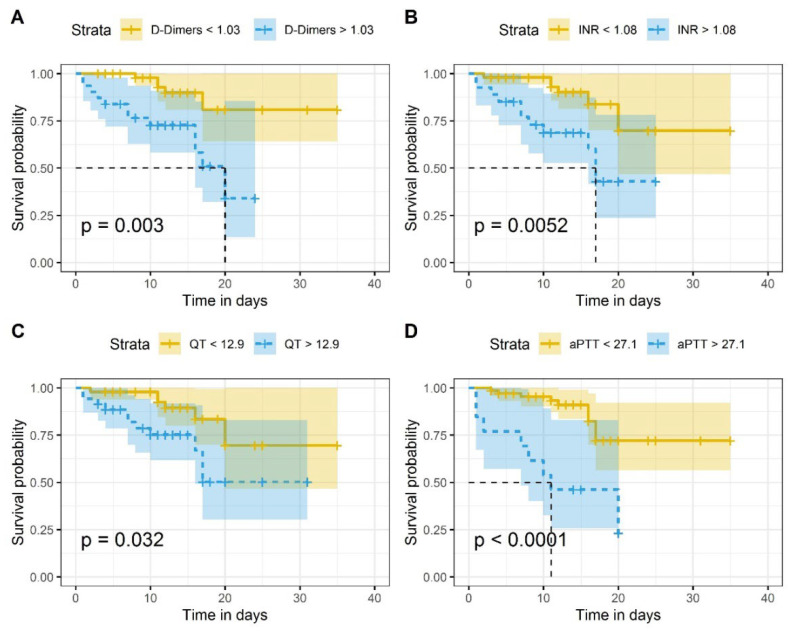
Kaplan–Meier survival curves of COVID-19 in-patients: (**A**) in accordance with established D-Dimer cutoff values (1.03 μg/mL); (**B**) in accordance with established INR cutoff values (1.06); (**C**) in accordance to established PT cutoff values (12.9 s); (**D**) in accordance with established aPTT cutoff values (27.1 s). On the x-axis is represented the time in days and on the y-axis the survival probability. The *p*-values show that the survival distributions are statistically significantly different. The null hypothesis tested is that there is no difference in the distribution of overall survival between groups (with coagulation biomarker levels above or below cut-off values) in the study population.

Figure 3 illustrates the Kaplan–Meier survival curve plotted in terms of the cut-off values of D-Dimer, INR, PT and aPTT. Differences in the survival of patients with coagulation biomarker levels above cut-off values in comparison to subjects with levels lower than cut-off values were statistically significant (Figure 3). The survival distributions for coagulation biomarker values above or below the set cut-off values were statistically significantly different: χ^2^ = 8.8 (*p* = 0.003) for D-dimer, χ^2^ = 7.8 (*p* = 0.005) for INR, χ^2^ = 4.6 (*p* = 0.03) for PT, and χ^2^ = 16.6 (*p* < 0.0001) for aPTT, respectively.

A univariate regression analysis was conducted to determine the relationship between coagulation biomarkers and in-hospital mortality. All biomarkers included were significant predictors of mortality in the analysis (Table 3).

In addition, a multivariate logistic regression was performed to assess the discriminatory ability of D-dimer, INR, PT and aPTT (above or below cut-off values) as prognostic factors for mortality, adjusted for age, comorbidities and sex. The results showed an aOR of 6.05 for D-dimer above 1.03; 6.50 for INR above 1.08, 3.94 for PT above 12.9 and 11.7 for aPTT above 27.1 (Table 4).

## 4. Discussion

Coagulation abnormality appears to be an important complication in patients with COVID-19. Therefore, in this retrospective study, we analyzed the clinical and laboratory data of 82 patients from Municipal Emergency Clinical Hospital of Timisoara, Romania with COVID-19 which were collected at the time of admission. Our study, based on coagulation dysfunction, analyzes the mortality rate of COVID-19 patients, which was 20.7%, higher than in other studies conducted in Romania at different periods on COVID-19 patients where the mortality rate was 13.5% and 15.7% respectively [12,13]. This higher rate can be explained by the fact that the other studies included patients admitted earlier during the pandemic, when protocols issued by the health authorities in Romania required the admission of all COVID-19 positive persons, including asymptomatic or mildly ill individuals. These protocols were later changed recommending admission only for patients with moderate/severe disease. However, Tan et.al reported in a meta-analysis and systematic review that included patients from 17 countries a higher in-hospital mortality rate of 28.1% [14]. 

In our study, aPTT was the parameter with the highest prediction of mortality in COVID-19 patients, with an AUC of 0.753 and also had a good sensitivity and the highest specificity (92%). In addition, consistent with our findings, several studies showed that aPTT was significantly longer in non-surviving COVID-19 patients [15,16,17]. The second most significant parameter in the mortality of COVID-19 patients from our study is D-dimer, which showed a good performance with an AUC of 0.751. Additionally, in our findings it was observed that D-dimer had a high sensitivity (76%), the same as PT. Various studies have concluded that D-dimer has a better value in predicting patients who are likely to progress to severe cases [18,19]. Furthermore, in the meta-analysis performed by Zhao et.al, it is noted from their results that elevated D-dimer levels are an independent predictor of both mortality and complications [20]. In addition, Poudel et al. showed that the optimal cut-off value of D-dimer at admission for predicting mortality in patients with COVID-19 is1.5 μg/mL, similar to that reported in our study [21]. Moreover, in this study the D-dimer value at admission was an accurate biomarker for mortality prediction having an AUC of 0.807, which is also similar to our results [21]. Furthermore, some studies have shown that D-dimer levels are high in ICU patients, and a 70% increase in daily monitoring predicts in-hospital mortality during the medical ward stay [22]. However, Cidate et al. suggest that in patients with severe COVID19, D-dimer values do not accurately predict mortality rates and therefore should not be used for clinical decision making [23]. 

Another coagulation parameter evaluated in this paper was the INR, which also showed a good performance in predicting COVID-19 mortality with an AUC of 0.724. Similar to these findings, a meta-analysis study among patients with COVID-19 showed that elevated INR values, which suggest the present of systemic coagulopathy, were significantly linked to severe disease as well as increased mortality in COVID-19 patients [24]. Moreover, another significant coagulation parameter that we evaluated was PT, showing a good performance in our study with an AUC of 0.706 in predicting mortality among COVID-19 patients. Our findings are in concordance with the study conducted by Long et.al, where aPTT, D-dimer and PT had great value in disease prognosis [25]. However, in contrast to our findings, a study performed on 88 patients with COVID-19 showed that aPTT had no statistical difference in severe cases of COVID-19 [26]. 

This research has several limitations. One limitation of this research is that it has a retrospective design and was performed in a single medical clinic, with a small cohort size and without a control cohort due to the rapid emergence of COVID-19. The cohort may not have been sufficiently large enough to evaluate the predictive performance of coagulation parameters for in-hospital death, with only 17 deaths in this cohort. Second, the analysis of coagulation parameters was performed only at admission in all cases, with no laboratory data on dynamics during hospitalization. Third, the study did not assess serum levels of the von Willebrand factor, which is a clinically relevant biomarker.

## 5. Conclusions

In conclusion, our research highlighted the importance of assessing coagulation parameters in SARS-CoV-2 infection. Based on our findings, aPTT, D-dimer, PT and INR showed good performance in predicting the mortality of COVID-19 patients. Coagulation parameters may be beneficial for early recognition of patients at high risk of poor outcomes and can be used clinically to provide more aggressive care leading to reduced COVID mortality.

## Figures and Tables

**Table 1:** Characteristics of 82 COVID-19 participants.

Variables	Total	Survivors n = 65 (79.3%)	Non-Survivors n = 17 (20.7%)	*p*-Value
*Demographics*				
Age [Median (IQR)]	66.5 (17)	65 (13.91)	71 (12.13)	0.01
Gender: Female [n (%)]	42 (51.2%)	33 (50.8%)	9 (52.9%)	0.87
*Comorbidities* [n (%)]				
Hypertension	53 (64.6%)	40 (61.5%)	13 (76.5%)	0.25
CKD	35 (42.7%)	26 (40.0%)	9 (52.9%)	0.33
COPD	14 (17.1%)	10 (15.4%)	4 (23.5%)	0.42
Cardiac disease	35 (42.7%)	24 (36.9%)	11 (64.7%)	0.03
Diabetes	32 (39.0%)	25 (38.5%)	7 (41.2%)	0.83
Cancer	12 (14.6%)	9 (13.8%)	3 (17.6%)	0.69
*Coagulation Biomarkers* *[Median (IQR)]*				
D-dimers (μ/mL)	0.79 (1.18)	0.65 (0.77)	1.95 (1.36)	0.001
INR	1.04 (0.18)	1.02 (0.15)	1.11 (0.38)	0.004
aPTT (seconds)	22.65 (4.7)	22.0 (3.95)	27.1 (5.90)	0.001
PT (seconds)	12.65	12.3 (1.6)	13.3 (3.1)	0.009
Fibrinogen (mg/dL)	567.12 (246.0)	563.71 (262.0)	609.72 (202.7)	0.97
*Clinical course*				
Length of hospitalization [Median (IQR)]	13 (6.75)	14 (5)	10 (12)	0.03
ICU admission [n (%)]	17 (20.7%)	7 (10.8%)	10 (58.8%)	<0.001

aPTT = activated partial thromboplastin time; AUC = area under the curve; COPD = chronic obstructive pulmonary disease; CKD = chronic kidney disease; ICU, intensive care unit; INR = International Normalized Ratio; PT = pro-thrombin time. Coagulation biomarker normal ranges: D-dimer <0.50 μg/mL; INR = 0.8–1.18; aPTT < 40 s; PT = 10–14 s; Fibrinogen = 200–400 mg/dL.

**Table 2:** AUC-ROC of coagulation biomarkers and optimal cutoff.

Biomarker	Cut-Off	AUC	Youden	Sensitivity	Specificity
D-Dimer	1.03	0.751	0.472	76%	70%
INR	1.08	0.724	0.442	70%	71%
PT	12.9	0.706	0.380	76%	61%
aPTT	27.1	0.753	0.450	52%	92%

aPTT = activated partial thromboplastin time; AUC = area under the curve; INR = International Normalized Ratio; PT = pro-thrombin time.

**Table 3:** Univariate binominal logistic regression analysis.

Variables	Estimate	Std. Error	z-Statistic	*p* Value	Confidence Interval	
					Lower	Upper
D-Dimer > 1.03	1.76	0.59	2.94	<0.001	0.63	3.01
INR > 1.08	1.62	0.58	2.76	0.005	0.49	2.82
PT > 12.9	1.14	0.56	2.01	0.04	0.05	2.31
aPTT > 27.1	2.33	0.67	3.46	<0.001	1.04	3.72

aPTT = activated partial thromboplastin time; INR = International Normalized Ratio; PT = prothrombin time. Estimate (beta coefficient) = the log-odds of in-hospital death; z-statistic (Wald z-statistic) = the regression coefficient divided by standard error. The *p*-value for each variable tests the null hypothesis that the coefficient is zero.

**Table 4:** Multivariate binominal logistic regression analysis.

Variables	aOR	Std. Error	z-Statistic	*p* Value	Confidence Interval	
					Lower	Upper
D-Dimer > 1.03	6.05	0.72	2.50	0.01	1.57	27.5
INR > 1.08	6.50	0.67	2.81	<0.001	1.86	26.3
PT > 12.9	3.94	0.64	2.13	0.03	1.16	15.2
aPTT > 27.1	11.7	0.82	2.98	<0.001	2.53	68.9

aOR = adjusted Odds Ratio; aPTT = activated partial thromboplastin time; INR = International Normalized Ratio; PT = prothrombin time. Z-statistic (Wald z-statistic) = the regression coefficient divided by standard error. The *p*-value for each variable tests the null hypothesis that the coefficient is zero.

## Data Availability

The data sets used and/or analyzed during the present study are available from the correspondence author on reasonable request.
